# Hsa-miR-21-5p and Hsa-miR-145-5p Expression: From Normal Tissue to Malignant Changes—Context-Dependent Correlation with Estrogen- and Hypoxia–Vascularization-Related Pathways Genes: A Pilot Study

**DOI:** 10.3390/ijms26094461

**Published:** 2025-05-07

**Authors:** Mateusz Górecki, Aleksandra Żbikowska, Małgorzata Tokłowicz, Stefan Sajdak, Monika Englert-Golon, Mirosław Andrusiewicz

**Affiliations:** 1 Department of Cell Biology, Poznan University of Medical Sciences, Rokietnicka 5D, 60-806 Poznań, Poland; 86223@student.ump.edu.pl (M.G.); azbikowska@ump.edu.pl (A.Ż.); mtoklowicz@ump.edu.pl (M.T.); 2 Cell Biology Research Group, Student Scientific Society, Poznan University of Medical Sciences, Rokietnicka 5E, 60-806 Poznań, Poland; 3 Division of Gynecology, Poznan University of Medical Sciences, 10 Fredry St., 61-701 Poznań, Poland; ssajdak@ump.edu.pl; 4 Department of Gynaecology and Obstetrics, Collegium Medicum University of Zielona Góra, Zyty 28, 65-046 Zielona Góra, Poland; 5 Division of Gynaecological Oncology, Department of Gynaecology, Gynecological and Obstetrics Clinical Hospital, Poznan University of Medical Sciences, Polna 33, 60-535 Poznań, Poland; mgolon@ump.edu.pl

**Keywords:** ovarian cancer, microRNA, hsa-miR-21-5p, hsa-miR-145-5p, epigenetic biomarkers, estrogen-pathway-related genes, hypoxia–vascularization-dependent pathways

## Abstract

Ovarian cancer (OC) is a severe gynecological malignancy with a high mortality rate among women worldwide. It is often diagnosed at advanced stages due to the lack of effective screening methods. This study investigated the expression patterns of microRNAs (miRNAs) hsa-miR-21-5p and hsa-miR-145-5p as potential OC prognostic and diagnostic biomarkers and their correlation with estrogen-dependent (*ESR1* & *2*, *PELP1* and *c-SRC*) and hypoxia–neovascularization-induced (*HIF1A*, *EPAS1*, and *VEGFA*) pathway genes. Tissue samples obtained from twenty patients with confirmed ovarian cancer and twenty controls were analyzed using quantitative polymerase chain reaction (qPCR) to examine miRNA and mRNA levels. The qPCR analysis revealed significantly higher hsa-miR-21-5p and lower hsa-miR-145-5p expression in OC tissues than controls. Moreover, a significant trend was observed in hsa-miR-21-5p and hsa-miR-145-5p expression levels across normal, non-cancerous changes and malignant ovarian tissues. The hsa-miR-21-5p showed better diagnostic potential than hsa-miR-145-5p. We also observed inconsistent correlations in hsa-miR-21-5p and hsa-mir-145-5p and estrogen-related and hypoxia–neovascularization-dependent genes in ovarian cancer across all groups. This suggests that the relationship between these miRNAs and the selected genes is context-specific. Our findings suggest that hsa-miR-21-5p and hsa-miR-145-5p expression levels may be prognostic or diagnostic markers for ovarian cancer patients.

## 1. Introduction

Ovarian cancer (OC) is an aggressive gynecological malignancy with a high mortality rate among women worldwide. According to the World Health Organization (WHO), OC was the eighth most common cancer in women in 2020 and 2022, with over 324,000 new cases diagnosed annually. The mortality rate is similarly high, with OC accounting for almost 207,000 deaths in the same year [1,2,3]. In Poland, there were 4678 new cases of OC in 2022, with an age-standardized rate (ASR) per 100,000 of 12.2. The mortality rate was 3206, with an ASR per 100,000 of 7.0 [4]. These high rates result from the lack of a practical screening test with high sensitivity and specificity, resulting in the detection of over 70% of OC cases at an advanced stage [5,6].

The International Federation of Gynecology and Obstetrics (FIGO) staging system classifies OC into four stages based on the extent of tumor spread. Stage I OC is confined to the ovaries, while stage II involves minimal spread to adjacent pelvic structures. Stage III OC indicates more extensive pelvic or intra-abdominal spread, and stage IV OC represents distant metastasis, typically to the liver or lungs. The five-year survival rates for stages I, II, III, and IV decrease drastically with the stage (approximately 90%, 70%, 39%, and 17%, respectively), highlighting the importance of early detection [7,8,9].

Epithelial ovarian cancer (EOC) is the most common type of ovarian cancer, accounting for approximately 90% of all cases, and is classified into two main histologic subtypes. Type I EOC, also known as low-grade or low-malignant potential EOC, is characterized by a favorable prognosis and a relatively low risk of recurrence. This type comprises approximately 25% of all EOC cases. These tumors are generally slow-growing and are often diagnosed at an early stage (FIGO stages I or II). Type II EOC, also known as high-grade or high-malignant potential EOC, accounts for 75% of all EOC cases. These tumors are more aggressive and are typically diagnosed at later stages (FIGO stages III or IV). Type II EOC is associated with a poorer prognosis and a higher risk of recurrence [8,9].

A complex interplay of genetic and epigenetic changes underlies EOC’s development and clinical presentation. The most common genetic alterations in EOC involve mutations in the *BRCA1* and *BRCA2* genes. These genes are involved in DNA repair, and mutations in these genes lead to genomic instability and increased susceptibility to cancer [10]. Other frequently mutated genes in EOC include *TP53*, *KRAS*, and *BRAF* [11,12]. *TP53* is a tumor suppressor gene critical in regulating cell growth and apoptosis. Mutations in *TP53* can lead to uncontrolled cell proliferation and resistance to chemotherapy. *KRAS* and *BRAF* are oncogenes that promote cell growth and survival [10,11,12]. Mutations in these genes can lead to the development of aggressive EOC tumors. However, the role of these genes in EOC pathogenesis is well known [9,13].

Beyond genetic alterations, metabolic pathway modifications, and enzymatic changes, tumorigenesis and metastasis can also be associated with the modulation of microRNA (miRNA) expression [14]. MiRNAs are small, non-coding RNAs that regulate gene expression by targeting mRNAs for degradation or translational suppression [15,16].

Several miRNAs have been implicated in OC development and progression [17], and they are associated with effects on cellular processes. For instance, hsa-miR-21 promotes tumor growth and metastasis by targeting tumor suppressors [18,19,20,21,22,23]. Conversely, hsa-miR-628-5p, hsa-miR-34c-5p, hsa-miR-136, and hsa-miR-145-5p inhibit tumorigenesis by targeting genes involved in cell proliferation, migration, apoptosis induction, and chemotherapeutic sensitivity, and are additionally involved in survival and stemness suppression [17,24,25,26,27].

Estrogen- and hypoxia–vascularization–related pathway genes refer to a group of genes that are involved in processes regulated by estrogen and those that respond to hypoxia, particularly in the context of tumor biology and vascularization [28,29,30,31,32]. Estrogen is a key hormone that plays a significant role in the reproductive system and has various effects on many tissues throughout the body. In the context of cancer, particularly hormone-dependent cancers like some ovarian and breast cancers, estrogen can promote tumor growth [32,33]. Pathway genes involved in estrogen signaling typically include estrogen receptors (ESR1 and ESR2). These are proteins that mediate the effects of estrogen. They can influence gene expression and cellular behavior when activated by estrogen [32]. The regulatory proteins, such as proline-, glutamic acid-, and leucine-rich protein 1 (PELP1) and proto-oncogene tyrosine-protein kinase c-Src (SRC), can modify cell signaling pathways influenced by estrogen, leading to proliferation, differentiation, or survival of the cancer cells [34,35]. Low-oxygen conditions within tissues can occur in solid tumors due to rapid growth outpacing the blood supply (and therefore oxygen). Tumor cells adapt to these conditions to survive and thrive [36]. In response to hypoxia, cells activate specific signaling pathways that promote the formation of new blood vessels from pre-existing ones, which is vital for tumors to receive sufficient nutrients and oxygen, by modulating the expression of hypoxia–vascularization-related pathway genes [37,38,39]. Key genes in this pathway include *HIF1A* (hypoxia-inducible factor 1-alpha)—a transcription factor that is activated under low-oxygen conditions and regulates the expression of genes involved in angiogenesis and metabolism—*VEGFA* (vascular endothelial growth factor A)—a major regulator of angiogenesis; it promotes the growth of blood vessels and is often upregulated in tumors—and *EPAS1* (endothelial PAS domain protein 1)—another member of the HIF family that contributes to the cellular response to hypoxia [40,41,42].

Genes related to estrogen pathways, including *ESR1* and *ESR2*, their coregulator *PELP1*, and the *SRC*, play a role in the induction and progression of ovarian cancer. The dysregulation of the estrogen-dependent signaling pathway appears to be a critical factor linked to the pathology of ovarian cancer. Specific estrogen-mediated functions in the ovary and ovarian-derived cancers may arise from differing local interactions between estrogens and their receptors and coregulators [5]. It was postulated that the estrogen-related pathway gene expression mentioned above can be regulated directly or indirectly by hsa-miR-21 [43,44,45,46,47,48,49,50] and hsa-miR-145 [51,52,53,54,55,56]. Previous studies also investigated the differences in mRNA levels of hypoxia-inducible factor 1-alpha, endothelial PAS domain protein 1 (also known as hypoxia-inducible factor 2-alpha, *HIF2A*/*EPAS1*), and vascular endothelial growth factor A among cancerous tissue, benign hyperplastic changes in the ovary, and normal tissue, but also in other endometrial cancer [30,57]. In upstream or downstream expression control for hypoxia-induced and neovascularization-dependent pathways genes, the roles of hsa-miR-21 [58,59,60,61,62,63] and hsa-miR-145 [63,64,65] were also shown. In both case-to-case and case-to-control studies, disruptions in the expression levels of mRNA interdependent genes were observed [5,57]. Our results suggest that the mutual association in the expression carries prognostic significance for OC patients [5,57].

Understanding the role of miRNAs in the context of estrogen-related and hypoxia–neovascularization-dependent genes’ expression could be one of the key elements for developing effective therapeutic strategies to address this issue and improve patient outcomes. MiRNA-based therapies are promising for personalized treatment approaches targeting specific miRNA alterations associated with chemotherapy resistance in ovarian cancer patients. Still, the differential expression of miRNAs can serve as potential non-invasive biomarkers for ovarian cancer in the early stages of tumor progression. It could enable earlier and more effective treatment implementation, potentially improving patient survival and quality of life [14].

Ovarian cancer is a highly aggressive and often fatal malignancy with limited treatment options. Early detection and diagnosis are crucial for improving patients’ chances of survival. Different miRNAs have been shown to be promising biomarkers for various cancers, including ovarian malignancies. In this exploratory pilot study, we aimed to investigate hsa-miR-21-5p and hsa-miR-145-5p expression patterns in OC to identify potential connections with genes related to estrogen and hypoxia–vascularization pathways. This study is an initial step toward a more comprehensive examination of epigenetic gene regulation in cancer-related pathways. Thus, primarily, our study aimed to investigate the expression of hsa-miR-21-5p and hsa-miR-145-5p to evaluate their potential as biomarkers in ovarian tumorigenesis. Additionally, we investigated the correlation of estrogen-related pathway genes (*ESR1*, *ESR2*, *PELP1*, and *c-SRC*) with hsa-miR-21 and hsa-miR-145 in non-affected ovary and ovarian cancer. Also, we examined the association of these miRNAs and their potential hypoxia–neovascularization-dependent targets’ (*HIF1A*, *HIF2A*/*EPAS1*, and *VEGFA*) expression levels in ovarian malignancies. Our study shows inconsistent correlations across all groups, suggesting that the relationship between these miRNAs and the selected genes is context-specific. These varying correlations highlight the potential influence of individual patient characteristics and disease stage on the functional interplay between miRNAs and genes related to estrogen-related and hypoxia-induced neovascularization signaling pathways.

This study aims to provide initial insights into the expression patterns of hsa-miR-21-5p and hsa-miR-145-5p in ovarian cancer (OC) and their potential associations with genes related to estrogen and hypoxia–vascularization pathways. We conducted a thorough review of the existing literature on hsa-miR-21-5p and hsa-miR-145-5p. We discovered 17 papers focusing on hsa-miR-21-5p, primarily highlighting its role in chemoresistance, cancer progression, and aggressive phenotypes, with connections to increased angiogenesis and poor survival outcomes. For hsa-miR-145-5p, we identified 20 papers discussing its role as a tumor suppressor linked to various signaling pathways, including its significant association with FIGO stages and overall prognosis in ovarian cancer.

Our study could contribute to developing novel prognostic or diagnostic tools and therapeutic strategies for ovarian cancer. Further studies are necessary to clarify the mechanisms underlying these observed correlations and their implications for ovarian pathophysiology.

## 2. Results

### 2.1. Patient Data

The groups of patients with histological cancer were as follows: serous carcinoma (*n* = 12, FIGO: IC *n* = 2; IIIA *n* = 1; IIIB *n* = 1; IIIC *n* = 7, IV *n* = 1, G3), undifferentiated (*n* = 1, FIGO IV, G3), clear cell (*n* = 1, FIGO IC, G3), epithelial-stromal Brenner tumors (*n* = 2, FIGO: IIIA G1, IIIB G2), mucinous adenocarcinomas (*n* = 2, FIGO: IIIB G1), squamous cell carcinoma (*n* = 1; FIGO IV), folliculoma (*n* = 1). The patients and controls were age-, body-mass-, and BMI-matched (Table 1). The analyzed patients were further also divided into samples without pathological changes (*n* = 10; ovary without changes; OWC), tissues in which the changes were in a benign stage and were not neoplastic (*n* = 10; benign ovarian changes; BOC), and tissues in which the changes were neoplastic (patients matching *n* = 20; Table 1).

In this case–control study and the subdivision into three groups (OWC, BOC, and patients), only a difference was observed in coexisting tumor numbers.

### 2.2. MiRNA Expression Changes in Analyzed Groups

**Figure 1:**
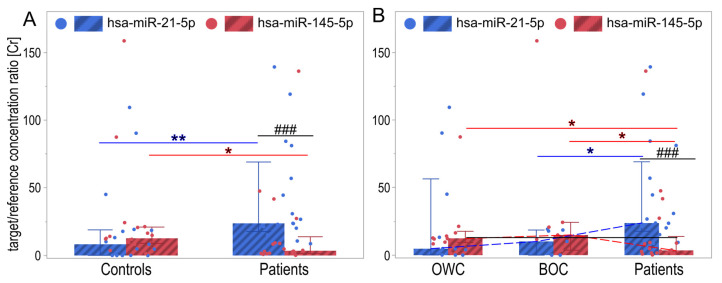
Box-and-whisker plot of normalized relative expression level [Cr] of hsa-miR-21-5p and hsa-miR-145-5p in (**A**) controls and the patient group, and (**B**) tissue samples without changes (OWC), with benign non-malignant changes (BOC), and neoplastic ovary patients. * *p* < 0.05, ** *p* < 0.01, ^###^ *p* < 0.001. Blue and red solid lines represent differences between hsa-miR-21-5p and hsa-miR-145-5p, respectively. The solid black line represents differences between both miRNA levels and dashed lines—a significant Jonckheere–Terpstra trend.

The analysis of miRNA levels in the control group (which included both unchanged tissues and benign non-neoplastic lesions) and cancerous tissues revealed significantly lower hsa-miR-21-5p expression in the control group compared to patients (*p* = 0.0014) and significantly higher hsa-miR-145-5p (*p* = 0.0268) (Figure 1A, Table 2). Additionally, in the controls, although the level of hsa-miR-21-5p was slightly lower than that of hsa-miR-145-5p, no significant differences were found (*p* = 0.2313). In the patient group, the expression level of hsa-miR-21-5p was significantly higher than that of hsa-miR-145-5p (Figure 1A, Table 2).

The analysis revealed significantly reduced hsa-miR-21-5p and increased hsa-miR-145-5p expression in control tissues (including unchanged and benign lesions) compared to cancerous tissues (*p* < 0.05; Table 2).

Comparative analysis of miRNA expression in three different tissue types (from non-pathologically altered to benign non-neoplastic lesions and ovarian cancer) showed significant differences in hsa-miR-21-5p expression between benign non-neoplastic lesions and ovarian cancer (*p* = 0.048). Additionally, an upward trend in the expression of this miRNA was observed in all three types of analyzed tissue (*p* = 0.009). In the case of hsa-miR-145-5p, significant differences in expression were found between normal ovary and ovarian cancer (*p* = 0.0425) and between benign non-neoplastic lesions and ovarian cancer (*p* = 0.0425). The expression trend of hsa-miR-145-5p is characterized by an initial increase and a subsequent decrease (*p* = 0.028). It is worth noting the significantly increased expression of hsa-miR-21-5p in women with ovarian cancer compared to hsa-miR-145-5p levels (*p* = 0.0002; Table 2, Figure 1B). On the other hand, hsa-miR-145-5p, compared to hsa-miR-21-5p, shows slightly higher expression (but not significantly) in unchanged tissue and benign non-neoplastic lesions in the ovary (Table 2, Figure 1B).

The posteriori power estimation for hsa-miR-21-5p, with sample sizes of 20 cases in each group, shows a power of approximately 0.87 (or 87%), which is generally considered good. There is a relatively low risk (13%) of type II errors. The power of hsa-miR-145-5p with the same sample size was approximately 0.63 (or 63%). A power of 0.63 is considered moderate; while not ideal, it is not exceptionally low. There is a relatively high risk (37%) of a type II error.

**Figure 2:**
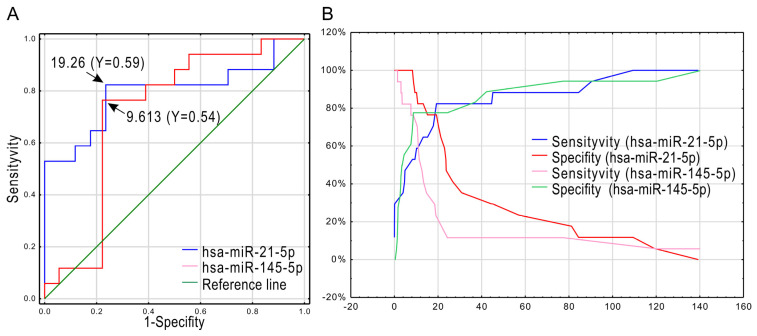
Receiver operating characteristic curves (ROC) and sensitivity/specificity analysis of hsa-miR-21-5p and hsa-miR-145-5p. (**A**) ROC curves for hsa-miR-21-5p (blue line) and hsa-miR-145-5p (red line). Cut-off points were established using Youden’s J statistic (Y). Based on Youden’s *J* Statistics for hsa-miR-21-5p, the cut-off = 19.26, Y = 0.59, and hsa-miR-145-5p = 9.163, Y = 0.54. (**B**) Sensitivity and specificity plots. Sensitivity is indicated by the blue (hsa-miR-21-5p) and red (hsa-miR-145-5p) lines; specificity is represented by the green (hsa-miR-21-5p) and purple (hsa-miR-145-5p) lines.

We performed receiver operating characteristic (ROC) curve analysis and sensitivity/specificity analysis of hsa-miR-21-5p and hsa-miR-145-5p (Figure 2A,B). Based on Youden’s *J* index (Y), hsa-miR-21-5p (destimulant), and hsa-miR-145-5p (stimulant), the cut-off points for the expression level of both miRNAs were estimated. For hsa-miR-21-5p, the cut-off was = 19.26, Y = 0.59 (good test, suitable for use), and for hsa-miR-145-5p = 9,163, Y = 0.54 (moderate test, limited application). Sensitivity and specificity plots for hsa-miR-21-5p and hsa-miR-145-5p across a range of thresholds show that hsa-miR-21-5p demonstrated higher sensitivity at higher thresholds but a corresponding decrease in specificity. In contrast, hsa-miR-145-5p showed a more balanced relationship between sensitivity and specificity across the threshold range (Figure 2B).

Considering the Youden index, hsa-miR-21-5p showed better diagnostic values than hsa-miR-145-5p.

### 2.3. MiRNA and mRNA Expression Correlation

Spearman’s rank correlation analysis was performed across four groups: all cases, controls, patients with ovarian pathologies, ovaries without changes (OWC), and those with benign changes (BOC). We investigated the correlations between the expression levels of hsa-miR-21-5p and hsa-miR-145-5p and a panel of genes implicated in estrogen-related and hypoxia-induced neovascularization pathways. The results, detailed in Table 3 and Table 4, reveal complex and context-dependent relationships, with correlations varying depending on the specific miRNA, gene, and patient group.

#### 2.3.1. Hsa-miR-21-5p Correlations

Estrogen-related genes. The correlations between hsa-miR-21-5p and estrogen receptor 1 *ESR1*, *PELP1*, and *SRC* were statistically insignificant across groups, highlighting a lack of robust association. We observed a tendency (*p* = 0.0737) for moderate hsa-miR-21-5p and *ESR2* correlation in the control group. This correlation was significantly positive and strong in the OWC group, suggesting potential functional interaction in unchanged tissue. Although the *SRC* correlation was insignificant, there was also a tendency (*p* = 0.0897) towards a moderate and negative correlation (Table 3).

Hypoxia–neovascularization-related genes *HIF1A*, *HIF2A*, and *VEGF* showed mostly non-significant correlations with hsa-miR-21-5p. Nevertheless, in controls, a tendency (*p* = 0.0752) was observed toward a moderate positive correlation for *HIF1A* and a similar tendency (*p* = 0.0739) for *HIF2A* in tissue without changes. A significant and strong positive correlation for *HIF1A* was revealed in the group with benign, non-malignant changes. The hsa-miR-21-5p expression level was significant, positive, and moderately correlated with the expression of *VEGFA* in all cases and strongly in the ovary without changes (*p* = 0.0069 and *p* = 0.0033, respectively; Table 3).

#### 2.3.2. Hsa-miR-145-5p Correlations

Estrogen-related genes. hsa-miR-145-5p displayed a more complex correlation pattern with estrogen-related genes. No significant correlation was shown in *ESR1* and *SRC* with hsa-miR-145-5p in the all-cases group and subgroups. In contrast, *ESR2* displayed a significant (*p* = 0.0258), positive, and weak correlation in all cases and a significant (*p* = 0.0130), positive, and moderate correlation in the patient group. The correlation of hsa-miR-145-5p was the most noticeable in the case of *PELP1.* Its expression was significantly (*p* = 0.0085), moderately, and negatively correlated in controls, and significantly (*p* = 0.0039), moderately but positively correlated in the patient group, indicating possible regulatory differences between healthy and diseased states. Both OWC and BOC groups showed a trend (*p* < 0.1) toward negative and moderate correlation (Table 4).

**Hypoxia–neovascularization-related genes.** Correlations with hypoxia–neovascularization-related genes were inconsistent across groups for hsa-miR-145-5p. *HIF1A* exhibited a tendency (*p* < 0.1) towards weak, positive correlation in all cases and a strongly positive correlation in benign and malignant tissues, suggesting a potential influence on hypoxic response. *HIF2A* showed a significant (*p* = 0.0252) moderate and positive correlation in patients. No significant correlation was demonstrated for *VEGFA* among all groups (Table 4).

In the case of hsa-miR-21-5p and estrogen-related genes, mostly non-significant correlations were observed. A notable exception was a significant positive correlation between hsa-miR-21-5p and *ESR2* in the OWC (ovaries without changes) group, suggesting a potential functional interaction in healthy ovarian tissue. There was also a tendency towards a negative correlation with *SRC*. Regarding hsa-miR-145-5p, it showed a more complex pattern. There was no significant correlation between *ESR1* and *SRC* across the groups. However, a significant positive correlation with *ESR2* was observed in all cases and in the patient group. A significant negative correlation with *PELP1* was found in the controls and a significant positive correlation was found in the patient group, highlighting a potential regulatory difference between healthy and diseased states.

There were mostly non-significant correlations in hypoxia–neovascularization-related genes, except for a strong positive correlation of hsa-miR-21-5p with *VEGFA* in all cases and especially in the OWC group, and a weak positive correlation with *HIF1A*. A tendency towards a positive correlation of hsa-miR-21-5p and *HIF2A* was observed in the OWC group. A significant positive correlation was found between hsa-miR-145-5p and *HIF2A* in the patient group. A tendency towards a positive correlation with *HIF1A* was observed in several groups. No significant correlations with *VEGFA* were found.

We did not observe a clear pattern in correlations of hsa-miR-21-5p and hsa-mir-145-5p and estrogen-related (*ESR1*, *ESR2*, *PELP1*, *SRC*) and hypoxia–neovascularization-dependent genes (*HIF1A*, *HIF2A*/*EPAS1*, and *VEGFA*) in ovarian cancer across all groups, suggesting that the relationship between these miRNAs and the selected genes is context-specific.

## 3. Discussion

The diagnosis of ovarian cancer remains a significant challenge in modern medicine. The statistical data indicate that 70% of cases are detected in advanced stages, which is associated with a poorer prognosis for patients. MiRNAs are proposed as potential biomarkers for ovarian cancer in the early stages of tumor progression [66]. According to the databases used, the miRNAs analyzed regulate seven common genes. However, the ovarian cancer regulation network is much more complicated.

MiRNAs have been shown to play a significant regulatory role in the progression, metastasis, and chemoresistance of ovarian cancer by acting directly on the tumor core, which consists of cancer stem cells (CSCs). Oncogenic miRNAs have been shown to be overexpressed in ovarian cancer, directly interacting with signaling pathways responsible for tumor initiation and progression, further contributing to its malignancy. In contrast, suppressor miRNAs were characterized by decreased expression in OC, thus stimulating the process of carcinogenesis [17].

In this study, we analyzed the levels of two other miRNAs, namely hsa-miR-21-5p and hsa-miR-145-5p, in tumor tissue samples from patients compared to the control group.

Previous studies on hsa-miR-21-5p have shown that its elevated expression level correlates with the FIGO stage and survival of patients with EOC [18]. Moreover, another study not only confirmed the correlation with FIGO but also added the degree of malignancy and metastasis of ovarian cancer. The same team also showed that a higher expression level of this miRNA leads to a decrease in the expression of the phosphatase and tensin homolog, a tumor suppressor protein [19].

Chan et al. [67] demonstrated that hsa-miR-21-5p plays a crucial role in cancer progression and drug resistance. Elevated hsa-miR-21 levels in ovarian cancer were associated with metastasis and poor prognosis. Their study shows that inhibiting hsa-miR-21-5p enhances ovarian cancer cell sensitivity to cisplatin. Conversely, hsa-miR-21 overexpression reduces cisplatin sensitivity and disrupts apoptosis by modulating tumor suppression and increasing the apoptosis inhibitor, contributing to chemotherapy resistance [67].

Researchers have shown that hsa-miR-145-5p is an ideal candidate for the EOC biomarker due to the already decreasing expression level of this molecule in the early stages of cancer development compared to its advanced stages [24]. Other researchers also observed its increased level in ovarian cancer patients compared to controls [68]. This observation is supported by studies showing that the retrogene *UTP14c* may promote TP53 degradation. Reduced TP53 levels could allow damaged cells to survive and potentially become cancerous. Additionally, it might impair the production of hsa-miR-145-5p, leading to unchecked cell proliferation and invasion [69]. It was also observed that the elevated level of the studied miRNA in EOC can significantly inhibit proliferation and migration processes and induce pro-apoptotic processes. The decreased expression level of hsa-miR-145-5p has also been associated with increased invasiveness, tumor growth, and increased expression proteins, which affect proliferation and migration processes [70,71,72,73].

Further studies show that overexpression of hsa-miR-145-5p inhibits cancer cell proliferation, colony formation, and tumor growth both in vitro and in vivo. These effects are mediated by suppressing *HIF1A* and *VEGF* [74]. *HIF1A* was also observed to be downregulated, and *VEGF* upregulated in malignant ovarian tumors [57]. Additionally, *p70S6K1* inhibits hsa-miR-145, preventing transcription factors from binding to the mRNA. This disruption leads to the overexpression of N-cadherin and the formation of multicellular spheroids. The elevated transcription factors and N-cadherin levels contribute to metastasis and tumor growth and are associated with poor survival in late-stage patients [75].

The relationship between hsa-miR-21-5p and other molecules of the estrogen-related pathway could be of a direct or indirect nature [43,44,45,47,48,49,50,76,77]. On the one hand, we did not observe a significant correlation between this miRNA and *ESR1*, *PELP1*, and *SRC.* The correlations had only a tendency to be moderate and negative in benign, non-cancerous affected samples of the ovary. On the other hand, the expression of hsa-miR-21-5p and *ESR2* was significantly, positively, and strongly correlated in normal tissue samples but not in malignant tissue. This suggests its potential role in the expression of *ESR2* regulation in non-affected tissue. Several studies examined the interaction between hsa-miR-21 and estrogen signaling pathways. In some instances, hsa-miR-21 is upregulated in estrogen-receptor-positive cancers, suggesting a possible influence on estrogen-dependent processes, but mainly indirectly, via other molecules [43,44,45,47,48,49,50]. A correlation was also found between hsa-miR-21-5p and *SRC*, particularly in cancer cell invasion and metastasis [76].

Additionally, another miRNA—hsa-miR-34a—acts as a tumor suppressor, post-transcriptionally downregulating *CD24* and *SRC*, which consequently decreases hsa-miR-21-5p expression and increases the expression of *PDCD4* and *PTEN* [77]. Furthermore, the regulation of hsa-miR-21 was influenced by other molecules, forming an intricate network impacting tumorigenesis [76,77]. However, more research is needed to fully understand this dynamic interplay. The exact interaction of hsa-miR-21-5p with *ESR1* (and other molecules like *ESR2* and *PELP1*) may vary based on cell type and specific conditions [43,44,45,48,49,50].

Similarly to our results, it was shown that hsa-miR-145-5p expression is frequently downregulated in other tumors and correlates with aggressive phenotypes [52,53,54,55,56]. The hsa-miR-145-5p interactions with estrogen receptors, PELP1, and SRC highlight its implications in, e.g., breast cancer progression and treatment [51,52,54,56]. We did not observe significant correlations between hsa-miR-145-5p and *ESR1* mRNA level. This is in line with other observations demonstrating that hsa-miR-145 directly targets the *ESR1* coding sequence, leading to decreased protein levels without affecting mRNA levels. This suggests a post-transcriptional mechanism of regulation. However, the effect was observed in ESR1-positive breast cancer cells and was linked to TP53 status (wild-type TP53 enhanced the effect) [51]. In our study, *ESR2* and *PELP1* were positively correlated with hsa-miR-145-5p in the patient group, and *PELP1* was also negatively correlated in controls. It was shown in other cancers that the mutual interplay of ESR2 and miRNA plays a role in cancer through the modulation of extracellular matrix components, which is one of the epigenetic mediators. Moreover, inhibiting hsa-miR-145 led to an aggressive phenotype, indicating hsa-miR-145 as a negative regulator of epithelial-to-mesenchymal transition [52]. We did not find any research focused on hsa-miR-145-5p and PELP1 expression, but it was shown that *PELP1* knockdown caused cytoskeletal defects and significantly affected the migratory potential of ovarian cancer cells [35]. Thus, as observed before, changes in the protein level of PELP1 [5] could be a result of hsa-miR-145-dependent epigenetic regulation. We did not observe a significant correlation between *SRC* and hsa-miR-145, but we do not exclude it.

**Figure 3:**
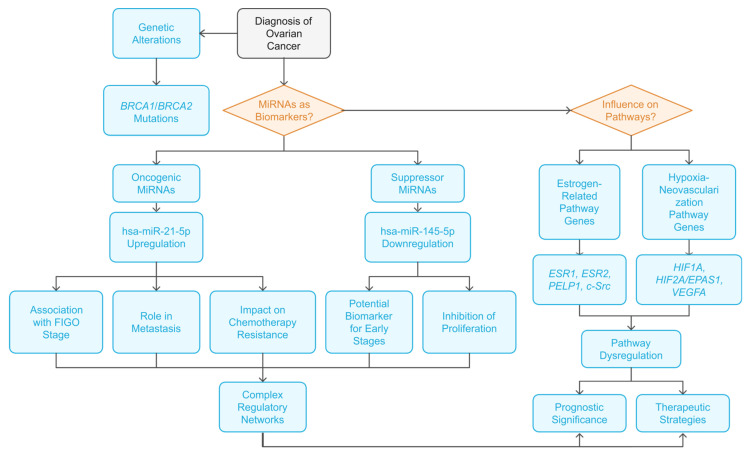
The flowchart of the roles of miRNAs in ovarian cancer diagnosis and pathway influences. Beyond the known genetic factors influencing ovarian cancer development, such as *BRCA1*/*BRCA2* mutations, other epigenetic factors, like miRNA expression level, can affect cancer manifestation and progression. The oncogenic or suppressor miRNAs are associated with cancer outcomes and could have implications for the dysregulation of different pathways.

MiRNA-21-5p was studied with established oncogenic roles in various cancers, and its dysregulation is linked to increased angiogenesis. Findings regarding hsa-miR-21-5p and its interaction with hypoxia-inducible factors HIF1A and HIF2A or its influence on VEGFA signaling highlight its impact on cancer progression and therapeutic implications [59,60,61,62,78,79]. Hsa-miR-21-5p acts as an oncogenic miRNA in this context by suppressing *HIF1AN*, thus indirectly activating the HIF1A/VEGF pathway and promoting tumorigenesis in choriocarcinoma under hypoxic conditions [61]. It appears to play a pro-angiogenic role, potentially influenced by hypoxic conditions, and the increased hsa-miR-21-5p might indirectly contribute to the increased VEGFA and HIF1A levels [78]. Several studies have demonstrated that hsa-miR-21-5p regulates the HIFs pathway. It can vary based on cancer type and cellular context. We observed a significant and strong (positive) correlation between *HIF1A* and hsa-miR-21-5p in benign, non-malignant changes. Our research aligns with other studies. It was shown that increased hsa-miR-21-5p maturation subsequently led to degradation of HIF1AN (HIF1A asparagine hydroxylase), an inhibitor of HIF1A, thus activating HIF1A-VEGFA signaling and promoting cancer progression [61]. The upregulation of *VEGFA*, downstream of hsa-miR-21-5p, strongly suggests that hsa-miR-21-5p contributes to the angiogenic response often triggered by hypoxic conditions [62]. In the case of *HIF2A*, we observed only a tendency toward a moderate and positive correlation in non-changed tissue. The *VEGFA* showed a significant, positive, and moderate correlation with hsa-miR-21-5p in all study cases, revealing concern for non-affected tissue. It was demonstrated that upregulation of hsa-miR-21-5p and downregulation of hsa-miR-100-5p activates the VEGFA pathway, leading to upregulation of mTOR, HIF1A, VEGFA, and MYC, which could stimulate endothelial survival, proliferation, and angiogenesis, and therefore could promote proatherogenic changes [62]. Also, significantly increased hsa-miR-21-5p levels in endothelial cell lines led to non-direct upregulation of VEGFA and CCND1, resulting in enhanced angiogenesis and vascular permeability. The studies demonstrate hsa-miR-21-5p’s multifaceted role in angiogenesis and cancer progression, showing its ability to activate pro-angiogenic pathways via direct targeting of genes and indirect modulation of signaling pathways (e.g., through regulation of HIF1A) (Figure 3). The regulation of hsa-miR-21-5p itself is complex, involving epigenetic mechanisms and transcription factors [59,60,61,62,78,79].

Multiple studies demonstrate that hsa-miR-145-5p plays a crucial role in regulating angiogenesis, often through its interaction with *HIF1A* and *HIF2A* and its influence on *VEGFA* expression. The relationship is usually inverse: decreased hsa-miR-145-5p levels are associated with increased HIF1A/HIF2A activity and VEGFA expression, promoting angiogenesis. Conversely, increased hsa-miR-145-5p expression inhibits these processes. The mechanisms involved frequently include direct gene targeting by hsa-miR-145-5p, affecting downstream pathways [63,64,65,80,81]. Our research showed a tendency for a strong, positive correlation of *HIF1A* and hsa-miR-145-5p in tissue characterized by benign changes. In the case of *HIF2A* in the patient group, the correlation with hsa-miR-145-5p was significant, moderate, and positive. In the case of *VEGFA*, the correlations were insignificant. Our observations regarding the expression level of hsa-miR-145-5p align with those of other researchers. Nevertheless, we could not find papers showing direct correlations with hypoxia-induced neovascularization-dependent pathway gene expression. Such regulation has only indirect roles [63,64,65,80,81]. Our studies highlight the significant involvement of miRNAs in angiogenesis and cancer progression, albeit within diverse tissue and cellular contexts.

Compared to the controls, the patient tissues were characterized by a higher expression of hsa-miR-21-5p and a lower expression of hsa-miR-145-5p. Thus, we observed statistically significant differences in their levels in patients. Moreover, it was demonstrated that the expression of hsa-miR-21-5p in three different tissue types (from non-pathologically altered to benign non-neoplastic lesions and ovarian cancer) shows an increasing trend, with a significant difference between benign non-neoplastic lesions and ovarian cancer. On the other hand, the same analysis of hsa-miR-145-5p showed an initially increasing and then decreasing trend. Statistically significant differences in expression were observed between normal ovaries and ovarian cancer and between benign non-neoplastic lesions and ovarian cancer. In both cases of miRNAs, our results confirm their oncogenic or tumor suppressor role in ovarian cancer.

Comparing groups with the absence or presence of comorbidities and coexisting cancers, no significant differences were observed in the level of the hsa-mir-21-5p. hsa-miR-145-5p showed a significantly lower level in women with coexisting tumors. Another study reveals the association of hsa-miR-21 expression and the radiation therapy response in cervical cancer. It suggests its potential utility, indicating patients with worse responses to therapy. In the same study, hsa-miR-145 demonstrated limited prognostic significance, likely due to its interactions with other cancer-related genes and pathways [82]. The combination of hsa-miR-21 and hsa-miR-145 did not substantially improve predictive accuracy, highlighting the need to investigate additional molecular markers to enhance its clinical application.

Research reports also indicate elevated levels of hsa-miR-21-5p functioning as an oncogene in colorectal cancer, glioma, prostate cancer, and lung cancer [20,21,22,23]. For instance, it targets tumor suppressor genes. Its overexpression also reduces their activity, enhancing cell proliferation and invasion in colorectal tumors. In gliomas, hsa-miR-21-5p downregulates *PDCD4* and *TIMP3*, facilitating tumor growth and resistance to apoptosis, making it a marker of poor prognosis. Elevated hsa-miR-21-5p levels also target genes metastasis suppressor genes, aiding lung cancer progression and invasion [83].

On the other hand, the expression of hsa-miR-145-5p acting as a suppressor in colorectal, breast, lung, or bladder cancers is significantly reduced, leading to carcinogenesis [84]. For instance, in colorectal cancer, hsa-miR-145-5p helps to regulate key oncogenic pathways, and its downregulation is associated with increased cancer cell proliferation and invasion. Similar suppressive roles have been observed in breast and bladder cancers, where its decreased expression correlates with enhanced tumor progression and metastasis [85]. The tumor-suppressive function of hsa-miR-145-5p involves the regulation of oncogenic targets, which play roles in cell proliferation and survival. If the hsa-miR-145-5p level decreases, these oncogenic factors can act out of control, promoting tumor growth and spread across different non-physiological locations [86].

Limitations of this study. This study included only 20 patients with ovarian cancer and 20 controls. This small sample size limits the generalizability of the findings. Larger studies are needed to confirm these results. The limited histological subtype of the ovarian cancer tissue samples obtained means that the results might not be generalizable across all subtypes, potentially limiting their clinical relevance. Our study found inconsistent correlations between the miRNAs and the genes involved in estrogen-related and hypoxia–neovascularization pathways. This inconsistency suggests the relationships are complex and context-dependent, requiring more in-depth investigation to elucidate the mechanisms. This study focuses on two miRNAs, which do not capture the full complexity of miRNA regulation in ovarian cancer. Further research is essential to confirm the utility of hsa-miR-21-5p and hsa-miR-145-5p as reliable biomarkers for ovarian cancer diagnosis and prognosis. There are unmeasured confounding factors, such as variations in patient demographics, underlying health conditions, or treatment histories, that could affect the expression levels of the miRNAs and their correlations with cancer progression.

## 4. Materials and Methods

### 4.1. Patients

Forty tissue samples were obtained from patients between 2019 and 2021 treated at the Division of Gynecological Oncology (Department of Gynecology) of the Gynecological and Obstetrics Clinical Hospital, Poznan University of Medical Sciences. All women were of Caucasian descent. Tissue samples with non-malignant histology obtained from patients who underwent total hysterectomy (*n* = 20) were qualified for the control group. The second group was the cancer-affected ovary samples (*n* = 20). Prior to surgery, each patient underwent a thorough interview, followed by a gynecological examination that included pelvic ultrasound imaging and a Doppler examination to document the presence of ovarian tumors. Blood analyses were conducted to determine the concentrations of CA-125, HE-4, and CEA antigens. During the gynecological examination, the following characteristics were assessed: the shape of the tumor, its consistency, mobility, location (unilateral or bilateral), and the presence of ascites. Based on the preoperative evaluations, the patients were qualified for laparoscopic removal of the ovarian tumor, which was performed by an experienced surgeon. Intraoperative macroscopic and microscopic pathological examinations determined the presence or absence of neoplastic changes. Tissue samples were sterilely transferred to the histopathology laboratory without fixation during the operation. The histopathologist selected a tumor tissue section for intraoperative examination based on macroscopic assessment. After confirming the presence of cancer cells during the intraoperative examination, a fragment of the tumor sample was fixed in a nucleic acid protection buffer for further analysis. The remaining surgical material was also fixed for a detailed histopathological evaluation postoperatively, which included determining the histological type, grade, and stage of the tumor. None of the patients underwent chemotherapy or radiotherapy prior to surgery. Samples obtained during surgery were immersed in RNA protective medium [87] and stored at −80 °C until nucleic acid isolation.

### 4.2. Methods

#### 4.2.1. High-Molecular-Weight RNA and MiRNA Isolation and Validation

Low-molecular-weight RNA (LMW RNA, containing miRNAs) and the high-molecular-weight RNA (HMW RNA) fraction were isolated separately from pulverized in liquid nitrogen 20–50 mg tissue samples using a double-column-based miRNA and RNA isolation system according to the manufacturer’s protocol (A&A Biotechnology, Gdańsk, Poland). In short, the cells were lysed in 800 µL of Fenozol (to deactivate endogenous RNases) and suspended by pipetting. Following a 5 min incubation at 50 °C and 200 rpm, 200 µL of chloroform (Avantor Performance Materials Poland S.A., Gliwice, Poland) was added, and the mixture was gently inverted five times. After a 3 min incubation at room temperature (RT), the samples were centrifuged at 12,000 rpm for 10 min at RT (benchtop centrifuge) to separate the phases. The 450 µL aqueous supernatant, containing both high- and low-molecular-weight (HMW and LMW) RNA, was transferred to a new Eppendorf tube. Then, 150 µL of isopropanol was added, mixed, and applied to a minicolumn. Subsequent centrifugations were performed at 12,000 rpm for 1 min at RT. To purify the LMW RNA, 400 µL of isopropanol was added to the flow-through and mixed. Then, 500 µL aliquots were repeatedly applied to a new minicolumn and centrifuged (the flow-through was discarded). Both minicolumns (containing HMW and LMW RNAs) underwent identical washing and elution procedures: two washes with 700 µL of wash solution and one wash with 200 µL, each followed by centrifugation. Finally, 100 µL of ultrapure water (Thermo Fisher Scientific, Waltham, MA, USA) was added to each minicolumn and incubated for 3 min (RT). The purified HMW and LMW RNAs were separately eluted by centrifugation [88,89]. The quantity, purity, and quality were analyzed spectrophotometrically. HMW RNA integrity was evaluated by agarose gel electrophoresis in denaturing conditions [89].

#### 4.2.2. MiRNA Reverse Transcription and Quantitative Polymerase Chain Reaction

Complementary miRNA DNA (cmiDNA) was synthesized in a four-step reaction using the TaqMan Advanced miRNA cDNA Synthesis Kit (Thermo Fisher Scientific, Waltham, MA, USA). Four nanograms of miRNA were used for the synthesis reaction. All steps were performed according to the manufacturer’s protocol and a previously described validated procedure [88]. The cmiDNAs were synthesized in duplicates for each sample and served as Quantitative Polymerase Chain Reaction (qPCR) templates.

The expression levels of hsa-miR-21-5p and hsa-miR-145-5p were determined by qPCR using the TaqMan MicroRNA Assay (Thermo Fisher Scientific, Waltham, MA, USA; Table 5). The reaction was carried out with SolisFAST Probe qPCR Mix with UNG (Solis Biodyne, Tartu, Estonia). The reaction mixture (20 μL in total) consisted of 1× qPCR master mix, one μL of TaqMan MicroRNA Assay probe, and 2.5 μL of cmiDNA diluted 1:10. To identify the best reference miRNA, we analyzed the expression of three miRNAs that we targeted as reference, namely, hsa-miR-26b-5p, hsa-miR-92a-3p, and hsa-miR-191-5p. The hsa-miR-191-5p was used as a reference miRNA characterized by the highest stability (also according to the miRNA tissue atlas: https://ccb-web.cs.uni-saarland.de/tissueatlas2/specificity, accessed on 8 March 2023). Additionally, hsa-miR-191-5p was proposed in the literature as one of the most suitable miRNAs in malignant samples [26], benign samples, and controls [90].

The thermal profile and acquisition steps were performed as per the SolisFast master mix protocol. The threshold cycles (Ct) mean values derived from replicated samples were used for further analysis. The efficiency estimation was described before [88]. Each miRNA sample and reference were efficiency-corrected, and miRNAs of interest were normalized to the hsa-191-5p reference. Cobas Z480 analyzer (Roche Diagnostics, Basel, Switzerland) and the dedicated software LCS480 1.5.1.62 SP3—UDF 2.1.0.26 (Roche Diagnostics) were used for the qPCR analyses. A relative expression level analysis was performed using the same software by comparing the expression level of the genes of interest with that of the reference gene (previously normalized by the efficiency factor). The resulting Cr (concentration ratio) value was used for further miRNA expression level determinations.

#### 4.2.3. Reverse Transcription and qPCR for Analyzed Genes

Complementary cDNA was synthesized using the Transcriptor Reverse Transcriptase protocol (Roche, Basel, Switzerland) in a 20 μL volume [5,89]. Quantitative PCR was performed using SolisFAST Probe qPCR Mix with UNG (Solis Biodyne, Tartu, Estonia). Primer sequences and TaqMan probe positions (Table 6) for the analyzed genes were designed using the Universal ProbeLibrary Assay Design Center (http://qpcr.probefinder.com, discontinued, accessed on 28 September 2017). The *HPRT* gene assay served as an internal control. As described earlier, QPCR cycling and acquisition steps were conducted in duplicates, using independently synthesized cDNA (20 μL total volume reaction) [5,89]. Mean values were used for statistical analyses, and reaction efficiencies were calculated from standard curves [89]. Analyses were performed on a Cobas Z480 analyzer (Roche Diagnostics) with LCS480 software (version 1.5.1.62 SP3—UDF 2.1.0.26; Roche Diagnostics). Efficiency-corrected relative expression levels were assessed by normalization to the *HPRT* reference. The concentration ratio (Cr) values were used for the statistical analyses.

### 4.3. Statistical Analyses

Statistical analyses were performed using Statistica version 13.3 (Dell Corp., Round Rock, TX, USA). The Shapiro–Wilk test was used to assess the conformity of the distribution of quantitative variables to the normal distribution. The *t*-Student’s test and one-way ANOVA tests (with Tukey’s post-hoc test) were applied for normally distributed data. For variables of non-parametric distribution, the Mann–Whitney *U* or Kruskal–Wallis tests (with Dunn Benjamini–Hochberg post-hoc test) were used to determine differences. The Jonckheere–Terpstra trend test was used for trend estimation in categorical variables. Median [lower quartile–upper quartile] (Me [Q1–Q3]) or mean ± standard deviation (*M* ± *SD*) were used to describe the experimental results. The distribution of categorical data was compared between different groups and subgroups with chi-square tests according to Cochran’s rules. Spearman’s rank correlation tests determined the correlation coefficient (R) between miRNA and mRNA expression levels. A posteriori power analysis was conducted. We used the Statistica software version 13.3—Power Analysis and Sample Size module to calculate the posterior sample size. We used the test for two independent means for two equal-sized samples with a one-tailed hypothesis about the difference in means. For the power calculation for each miRNA, we conducted separate calculations using the means for both samples—the experimental and control groups, with α set at 0.05, as well as a one-tailed hypothesis and a target power of 90%. The receiver operating characteristics (ROC) curve and Youden’s *J* statistic (Youden’s index) were used to analyze the discriminatory ability to distinguish cases and controls by miRNA expression level, selecting an optimal threshold value (cut-off point). The sensitivity and specificity of hsa-miR-21-5p and hsa-miR-145-5p were evaluated using expression raw data. A *p*-value < 0.05 was considered statistically significant.

## 5. Conclusions

The findings of this study suggest that elevated hsa-miR-21-5p expression and decreased hsa-miR-145-5p expression may serve as potential diagnostic markers for ovarian cancer. The altered expression patterns of these miRNAs could be used to differentiate between ovarian cancer and healthy tissues. The analysis revealed an increasing trend in hsa-miR-21-5p expression in all three analyzed tissue types (ovarian cancer, benign non-neoplastic lesions, and normal ovary). In contrast, hsa-miR-145-5p expression exhibited an initial increase followed by a decrease. hsa-miR-21-5p and hsa-miR-145-5p play differential roles in ovarian cancer development and progression. The altered expression patterns of these miRNAs could be used as potential biomarkers for ovarian cancer diagnosis and prognosis, and support the diagnostic process even at the early stages of pathological tissue differentiation. Due to the small sample size, further studies are needed to validate these findings and to investigate the functional mechanisms of hsa-miR-21-5p and hsa-miR-145-5p in ovarian cancer. The lack of consistency in correlations in hsa-miR-21-5p and hsa-mir-145-5p and estrogen-related (*ESR1*, *ESR2*, *PELP1*, *SRC*) and hypoxia–neovascularization-dependent genes (*HIF1A*, *HIF2A*/*EPAS1*, and *VEGFA*) in ovarian cancer across all groups suggests that the relationship between these miRNAs and the selected genes is context-specific. These varying correlations highlight the potential influence of individual patient characteristics and disease stage on the functional interplay between miRNAs and genes associated with estrogen-related and hypoxia-induced neovascularization signaling pathways. Further studies are necessary to clarify the mechanisms underlying these observed correlations and their implications for ovarian pathophysiology.

## Figures and Tables

**Table 1:** Characteristics of the study group and the control group.

Parameter		1st Division			2nd Division			
		Controls (*n* = 20)	Patients (*n* = 20)	*p*-Value	OWC (*n* = 10)	BOC (*n* = 10)	Patients (*n* = 20)	*p*-Value
Age [years] (M ± SD)		57 ± 11.5	61 ± 9.6	0.3296 ^#^	61 ± 10.8	54 ± 11.4	61 ± 9.6	0.1885 ^$^
Body mass [kg] (M ± SD)		72 ± 14.5	66 ± 16.0	0.2367 ^#^	69 ± 8.6	76 ± 18.9	66 ± 16.0	0.3163 ^$^
BMI [kg/m^2^] (M ± SD)		27.1 ± 6.16	25.9 ± 6.38	0.5484 ^#^	26 ± 3.52	28.4 ± 8.24	25.9 ± 6.38	0.5831 ^$^
Menopause [N]	Yes	13	17	0.2733 ^%^	7	6	17	0.3011 ^^^
	No	7	3		3	4	3	
Co-morbidities ^@^ [N]	Yes	11	12	>0.999 ^%^	7	4	12	0.3771 ^^^
	No	9	8		3	6	8	
Coexisting tumors [N]	Yes	6	16	0.0012 ^%,RR^	1	5	16	0.0001 ^^,BH^
	No	14	4		9	5	4	

Legend: M ± SD—Mean ± standard deviation, BMI—body mass index, OWC—ovary without changes, BOC—benign changes, non-cancerous ovary tissue, ^#^—*t*-Student’s test, ^$^—one way ANOVA, ^%^—Yates test, ^—Fisher’s exact test, ^@^—non-malignant, ^RR^—odds ratio with 95% confidence interval: RR = 9.33 [2.17–39.96], ^BH^—Benjamini–Hochberg post hoc for OWC vs. patients *p* = 0.0037.

**Table 2:** Descriptive statistics for the hsa-miR-21-5p and hsa-miR-145-5p concentration ratio in analyzed groups (patients and controls).

Group/miRNA	M ± SD	Me [Q1–Q3]	Min	Max
Controls/hsa-miR-21-5p	20.3 ± 32.2	8.3 [0.1–18.6]	0.0	109.4
Patients/hsa-miR-21-5p	43.5 ± 39.6	23.8 [20.2–57]	8.7	139.3
OWC/hsa-miR-21-5p	27.2 ± 40.9	4.7 [0.1–45.1]	0.0	109.4
BOC/hsa-miR-21-5p	10.6 ± 8.4	10.2 [0–18.6]	0.0	19.3
Controls/hsa-miR-145-5p	25.4 ± 39.3	12.7 [9.6–20.7]	1.4	158.6
Patients/hsa-miR-145-5p	16.9 ± 33.0	3.5 [1.6–9.3]	0.2	136.2
OWC/hsa-miR-145-5p	19.6 ± 24.4	12.4 [9.6–16.5]	1.4	87.5
BOC/hsa-miR-145-5p	33.8 ± 55.6	14.9 [3.7–24.3]	3.2	158.6

Legend: M ± SD—Mean ± standard deviation, Me [Q1–Q3]—median [lower-upper quartile], Min-Max—minimal and maximal concentration ratio values, OWC—ovary without changes, BOC—benign changes, non-cancerous ovary tissue.

**Table 3:** Spearman’s rank correlation coefficients for hsa-miRNA-21-5p level and estrogen-related pathways and hypoxia-induced neovascularization-dependent pathways genes expression.

Gene	All Cases (*n* = 40)		Controls (*n* = 20)		Patients (*n* = 20)		OWC (*n* = 10)		BOC (*n* = 10)	
	*R*	*p*-Value	*R*	*p*-Value	*R*	*p*-Value	*R*	*p*-Value	*R*	*p*-Value
*ESR1* ^a^	0.18	0.3100	0.01	0.9739	−0.19	0.4709	0.43	0.2129	−0.63	0.1289
*ESR2* ^a^	0.08	0.6547	0.44	0.0737	0.15	0.5649	0.78	**0.0084**	−0.27	0.5641
*PELP1* ^a^	−0.24	0.1748	−0.38	0.1379	0.34	0.1801	−0.12	0.7364	−0.59	0.1590
*SRC* ^a^	−0.23	0.1975	−0.14	0.5795	−0.13	0.6126	0.15	0.6761	−0.68	0.0897
*HIF1A* ^b^	0.03	0.8530	0.44	0.0752	0.10	0.7045	0.45	0.1869	0.77	**0.0408**
*HIF2A* ^b^	0.06	0.7541	0.21	0.4165	0.20	0.4450	0.59	0.0739	−0.16	0.7283
*VEGFA* ^b^	0.46	**0.0069**	0.15	0.5698	0.19	0.4565	0.67	**0.0033**	−0.45	0.3104

Legend: OWC—ovary without changes, BOC—benign changes, non-cancerous ovary tissue, *R*—*rho* Spearman’s rank correlation coefficients; *p*-values < 0.05 are indicated in bold, ^a^—estrogen-related pathway genes, ^b^—hypoxia–vascularization-related genes.

**Table 4:** Spearman’s rank correlation coefficients for hsa-miR-145-5p level and estrogen-related pathways and hypoxia-induced neovascularization-dependent pathways genes expression.

Gene	All Cases (*n* = 40)		Controls (*n* = 20)		Patients (*n* = 20)		OWC (*n* = 10)		BOC (*n* = 10)	
	*R*	*p*-Value	*R*	*p*-Value	*R*	*p*-Value	*R*	*p*-Value	*R*	*p*-Value
*ESR1* ^a^	−0.10	0.5654	−0.13	0.6291	0.13	0.6155	−0.07	0.8544	−0.14	0.7599
*ESR2* ^a^	0.38	**0.0258**	0.22	0.3879	0.57	**0.0130**	0.53	0.1157	−0.33	0.4736
*PELP1* ^a^	0.09	0.6125	−0.62	**0.0085**	0.64	**0.0039**	−0.60	0.0670	−0.67	0.0971
*SRC* ^a^	0.04	0.8081	−0.41	0.1005	0.25	0.3073	−0.48	0.1615	−0.36	0.4316
*HIF1A* ^b^	0.30	0.0852	0.39	0.1195	0.39	0.1093	0.22	0.5334	0.71	0.0713
*HIF2A* ^b^	0.22	0.2014	−0.12	0.6598	0.53	**0.0252**	0.08	0.8287	−0.25	0.5887
*VEGFA* ^b^	0.21	0.2361	0.05	0.8372	0.10	0.6987	0.28	0.4250	0.04	0.9394

Legend: OWC—ovary without changes, BOC—benign changes, non-cancerous ovary tissue, *R*—*rho* Spearman’s rank correlation coefficients; *p*-values < 0.05 are indicated in bold, ^a^—estrogen-related pathway genes, ^b^—hypoxia–vascularization-related genes.

**Table 5:** TaqMan hydrolytic probes for miRNA expression analysis.

miRNA Symbol	Assay Reference Number	Mature miRNA Sequence
hsa-miR-21-5p	(477952_mir)	CAACGGAAUCCCAAAAGCAGCUG
hsa-miR-145-5p	(477916_mir)	GUCCAGUUUUCCCAGGAAUCCCU
hsa-miR-191-5p	(000397_mir)	UAGCUUAUCAGACUGAUGUUGA

Catalog number: A25576; Thermo Fisher Scientific (Waltham, MA, USA).

**Table 6:** Quantitative polymerase chain reaction probes and primer designations.

Gene of Interest	Manufacturers Designation	Cat. No.	Primer Sequence 5′→3′		Amplicon Length [bp]
*ESR1*	#69 ^RD^	04688686001	F	ccttcttcaagagaagtattcaagg	160
			R	attcccacttcgtagcatttg	
*ESR2*	dHsaCPE5037392 ^BR^	10041596	*		87
*PELP1*	#24 ^RD^	04686985001	F	caaggaggagactcacaggag	131
			R	gcagcaggcagtagagttca	
*SRC*	#21 ^RD^	04686942001	F	gccatgttcactccggttt	100
			R	cagcgtcctcatctggtttc	
*HIF1A*	#71 ^RD^	04688945001	F	tttttcaagcagtaggaattgga	76
			R	ttccaagaaagtgatgtagtagctg	
*EPAS1*	#39 ^RD^	04687973001	F	gaaaacatcagcaagttcatgg	77
			R	cagggatgagtgaagtcaaagata	
*VEGFA*	#69 ^RD^	04688686001	F	cgaacgtacttgcagatgtga	88
			R	gagagatctggttcccgaaa	
*HPRT*	102,079 ^RD^	05532957001	*		

* Sequences are withheld as a trade secret of the manufacturer; F—forward primer; R—reverse primer; [bp] base pairs; manufacturers: ^RD^—Roche (Basel, Switzerland), ^BR^—BioRad (Hercules, CA, USA).

## Data Availability

The raw data supporting the conclusions of this article will be made available by the authors on request.
